# Trajectories of Adherence to Home-Based Exercise Recommendations Among People With Low Back Pain: A Longitudinal Analysis

**DOI:** 10.1093/ptj/pzad091

**Published:** 2023-09-05

**Authors:** Remco M Arensman, Martijn W Heymans, Corelien J J Kloek, Raymond J W G Ostelo, Cindy Veenhof, Tjarco Koppenaal, Martijn F Pisters

**Affiliations:** Center for Physical Therapy Research and Innovation in Primary Care, Julius Health Care Centers, Utrecht, The Netherlands; Physical Therapy Research, Department of Rehabilitation, Physiotherapy Science and Sport, Brain Center, University Medical Center Utrecht, Utrecht University, Utrecht, The Netherlands; Department of Data Science and Bio-Statistics, Amsterdam Public Health Research Institute, Amsterdam University Medical Centre, Location VUmc, North-Holland, Amsterdam, The Netherlands; Center for Physical Therapy Research and Innovation in Primary Care, Julius Health Care Centers, Utrecht, The Netherlands; Expertise Center Healthy Urban Living, Research Group Innovation of Human Movement Care, HU University of Applied Sciences, Utrecht, The Netherlands; Department of Health Sciences, Faculty of Science, VU University Amsterdam, Amsterdam Movement Sciences Research Institute Amsterdam, North-Holland, Amsterdam, The Netherlands; Department of Epidemiology and Data Science, Amsterdam University Medical Centre, Location VUmc, North-Holland, Amsterdam, The Netherlands; Center for Physical Therapy Research and Innovation in Primary Care, Julius Health Care Centers, Utrecht, The Netherlands; Physical Therapy Research, Department of Rehabilitation, Physiotherapy Science and Sport, Brain Center, University Medical Center Utrecht, Utrecht University, Utrecht, The Netherlands; Expertise Center Healthy Urban Living, Research Group Innovation of Human Movement Care, HU University of Applied Sciences, Utrecht, The Netherlands; Center for Physical Therapy Research and Innovation in Primary Care, Julius Health Care Centers, Utrecht, The Netherlands; Physical Therapy Research, Department of Rehabilitation, Physiotherapy Science and Sport, Brain Center, University Medical Center Utrecht, Utrecht University, Utrecht, The Netherlands; Research Group Empowering Healthy Behaviour, Department of Health Innovations and Technology, Fontys University of Applied Sciences, North-Brabant, Eindhoven, The Netherlands; Center for Physical Therapy Research and Innovation in Primary Care, Julius Health Care Centers, Utrecht, The Netherlands; Physical Therapy Research, Department of Rehabilitation, Physiotherapy Science and Sport, Brain Center, University Medical Center Utrecht, Utrecht University, Utrecht, The Netherlands; Research Group Empowering Healthy Behaviour, Department of Health Innovations and Technology, Fontys University of Applied Sciences, North-Brabant, Eindhoven, The Netherlands

**Keywords:** Adherence, Home-Based Exercise, Low Back Pain, Physical Therapists, Trajectories

## Abstract

**Objective:**

This study aimed to examine the presence of distinct trajectories of adherence to home-based exercise recommendations among people with low back pain (LBP). This study also aimed to identify differences in baseline characteristics among groups.

**Methods:**

This study was a secondary analysis of a prospective, multicenter cluster randomized controlled trial investigating the cost-effectiveness of a stratified blended physical therapist intervention compared to usual care physical therapy in patients with LBP. The intervention group received usual care with integrated support via a smartphone app. A total of 208 patients were recruited from 58 primary care physical therapist practices. Baseline data included patient characteristics, physical functioning, pain intensity, physical activity, fear avoidance, pain catastrophizing, self-efficacy, self-management ability, and health-related quality of life. The Exercise Adherence Scale (score range = 0–100) was used to measure adherence during each treatment session. Latent class growth analysis was used to estimate trajectories of adherence.

**Results:**

Adherence data were available from 173 out of 208 patients (83%). Data were collected during an average of 5.1 (standard deviation [SD] = 2.5) treatment sessions, with total treatment duration of 51 (SD = 41.7) days. Three trajectory classes were identified: “declining adherence” (12%), “stable adherence” (45%), and “increasing adherence” (43%). No differences in baseline characteristic were found between groups.

**Conclusion:**

Three adherence trajectories to exercise recommendations were identified in patients with LBP. However, baseline characteristics cannot identify a patient’s trajectory group.

**Impact:**

Despite the presence of distinct trajectories of adherence in patients with LBP, physical therapists should not attempt to place a patient in a trajectory group at the start of treatment. Instead, adherence should be closely monitored as treatment progresses and supported when required as part of an ongoing process.

## Introduction

The impact of low back pain (LBP) on society and health care and its related cost is well established.^1^ For decades, exercise has been studied as a potential treatment for LBP, and as a result, exercise (eg, strength training or mobility exercises) is part of the core recommendations for the treatment of LBP in many clinical guidelines.^2–5^ However, the heterogeneity of effects found between different studies, caused by factors such as differences in interventions, methodologies, and follow-up durations, makes it difficult to determine which exercise intervention is most effective for individual patients. Despite this, pooled data from 27 trials involving 3514 participants showed that exercise therapy reduces pain and functional limitations compared with non-exercise treatment in patients with persistent LBP.^6^ Furthermore, many interventions incorporate home-based exercise (HBE) to increase treatment effectiveness or as a solution to alleviate the burden of LBP on the public health system.^7^ However, the effectiveness of exercise interventions largely depends on adherence, and without supervision from a clinician, patient adherence to HBE recommendations is often low, reducing treatment effectiveness.^8–10^

The World Health Organization defined adherence as “the extent to which a person’s behavior – taking medication, following a diet, and/or executing lifestyle changes, corresponds with agreed recommendations from a health care provider.”^11^ Adherence to HBE recommendations would then be defined as “the extent to which a person’s behavior corresponds with agreed HBE recommendations from a health care provider.” Research has shown that adherence to exercise recommendations from a physical therapist is a complicated and multi-factorial construct, with factors such as social support, guidance by the therapist, the number of exercises, self-motivation, self-efficacy, and psychological aspects influencing individual patients’ adherence.^10^

To increase patient adherence to HBE recommendations, interventions targeting patient adherence were developed and showed varying levels of effectiveness. For instance, a trial investigating the effects of practitioner communication skills training on patients’ adherence to HBE recommendations in patients with chronic LBP found that adherence declined over time and the intervention appeared to only slow the rate of decline.^12^ In another study, using a smartphone application to support adherence to HBE recommendations increased self-reported adherence compared to usual care after 3 months.^13^ Unfortunately, the complexity of adherence to HBE recommendations makes it a challenging construct to measure resulting in a large number of different measurement instruments.^14^^,^^15^ Although many instruments aimed at measuring adherence to HBE recommendations are available, there is a lack of validated instruments making adherence difficult to study.^14^ To fill this gap, the recently developed Exercise Adherence Scale (EXAS) was designed to measure adherence to frequency, intensity, and quality of performance recommendations for HBE.^16^ The EXAS allows for the measurement of adherence during the treatment process, providing more detailed information on the patient’s self-reported adherence.

With the large number of both patient- and therapist-related factors influencing patient adherence, it is likely that adherence varies significantly between individuals and over time. Furthermore, the trajectory of adherence over time during the treatment period is likely to vary among patients with LBP.

Although, to date, no studies have examined the presence of common trajectories of adherence to HBE recommendations in patients with LBP, evidence for distinct trajectories of adherence has been found in patients with osteoarthritis of the knee and/or hip, and in older adults with cognitive impairment rehabilitating at home after hip fractures.^17^^,^^18^ Although both the nature of rehabilitation and the health of the patients are not comparable to those of patients with LBP, these studies showed that trajectories of adherence are present in different groups of patients. Each distinct trajectory has different clinical implications, and early identification of group membership of a patient can assist clinicians to determine which patients benefit from interventions designed to boost adherence and at what timepoint during treatment. Furthermore, identification of factors associated with the trajectory of adherence of patients with LBP can assist in the development of interventions to boost patient adherence. Therefore, investigating the unique trajectories of adherence to HBE recommendations from a physical therapist in patients with LBP has the potential to increase the effectiveness of interventions for this patient group.

Therefore, the aim of this study was to investigate the presence and proportion of groups of patients with distinct trajectories of adherence to HBE recommendations among people with LBP and to identify differences in baseline characteristics between groups.

## Methods

### Study Design

This study was a secondary analysis of a prospective, multicenter cluster randomized controlled trial investigating the cost-effectiveness of a stratified blended physical therapist intervention compared to usual care in patients with LBP. The detailed study protocol of the parent trial has been published previously.^19^ The Guidelines for Reporting on Latent Trajectory Studies checklist was used to aid in the reporting of this study.^20^

One hundred and twenty-two physical therapists (median 12; interquartile range 19.5 years of experience) from 58 primary care physical therapist practices in the Netherlands participated in the study and recruited patients from July 2018 to December 2019. Practices were cluster-randomized to either the intervention group or usual care group. The patients included in the parent trial were treated as a single cohort of patients with LBP and treatment group allocation was included in the analyses as a baseline characteristic. The study was approved by the Medical Research Ethics Committee of the University Medical Center Utrecht, the Netherlands (ISRCTN 94074203).

### Participants

Patients with LBP were recruited through the participating physical therapists. Prior to participating, written informed consent was obtained from all patients, and eligibility was checked by the researchers (R.A. or T.K.). A patient was eligible for participation when (1) the patient requested physical therapist treatment for LBP (pain in the lumbosacral region sometimes associated with radiating pain to the buttock or leg),^21^^,^^22^ (2) aged 18 years or older, (3) in possession of a smartphone or tablet with internet access, (4) B1-level proficiency in the Dutch language.^23^ Patients were excluded when patients had: (1) a specific cause of LBP determined through medical imaging or diagnosed by a medical doctor (including pelvic girdle pain caused by current pregnancy), or (2) serious comorbidities. When inclusion for the trial ended, a total of 208 patients enrolled in the study.

### Treatment

All patients received treatment based on the clinical guideline for LBP from The Royal Dutch Society for Physiotherapy.^22^ The guideline recommends giving information and advice about the nature and diagnosis of LBP, the course and prognosis of LBP, and inhibiting and facilitating factors. Furthermore, the guideline recommends providing personalized exercise therapy, and behavior-oriented and hands-on treatments for specific patients. Patients in the intervention group received stratified blended physical therapy, consisting of usual care face-to-face physical therapy with integrated support from a smartphone application (e-Exercise LBP).^19^^,^^24^ The content of the e-Exercise LBP app was also based on the clinical guideline for LBP from The Royal Dutch Society for Physiotherapy.^22^ The content of the e-Exercise application was tailored to the needs of the patient by the physical therapist and contained texts and videos with self-management information, the HBE exercises recommended by the physical therapist, and a module to support the patient’s physical activity. Each patient received treatment exclusively from the same physical therapist, maintaining consistent therapeutic interactions between patients and their respective physical therapists throughout the study duration. The evaluation of the effectiveness of the e-Exercise LBP intervention in patients with LBP showed no significant between-group differences after 3 months for almost all outcomes.^25^ Only fear-avoidance beliefs and self-reported adherence to prescribed HBE showed a statistically significant difference between the intervention and control groups. To account for the possible effect of the intervention on adherence during physical therapist treatment, treatment group allocation was included as a baseline characteristic for data analyses.

### Outcomes

All outcomes were measured at baseline only, except for adherence to HBE recommendations. Adherence to HBE recommendations was measured using the EXAS during patients’ visits at the clinic and recorded on a case report form by the physical therapist.^16^ During the first treatment session, the exercises and recommended frequency and intensity were recorded, and at the start of the following treatment session, the patient reported adherence to the recommendations. The physical therapist recorded patient-reported adherence using the EXAS and rated the quality of performance of the exercises on a 5-point scale (poor, moderate, reasonable, good, excellent). Adherence was then calculated as a percentage, and the resulting percentage was modified by the quality of performance rating. The EXAS score was then obtained by calculating the mean modified adherence percentage for all exercises, resulting in an EXAS score for every treatment session after the first session. The EXAS score ranges from 100 (perfect adherence) to 0 (no adherence). After the last treatment session, the therapist recorded the total number of treatment sessions.

For the comparison between groups with distinct trajectories of adherence, patients completed questionnaires on patient characteristics, physical functioning, pain intensity, fear avoidance, pain catastrophizing, self-efficacy, self-management ability, and health-related quality of life at the start of the study.

Physical functioning was measured using the Oswestry Disability Index (ODI), version 2.1a.^26^^,^^27^ The score on the ODI ranges from 0 to 100 with a higher score indicating increased functional disability. The ODI is part of the “Core Outcome Set” for research involving patients with nonspecific LBP.^28^

Pain intensity was measured with an 11-point Numeric Pain Rating Scale for the average pain intensity in the past 7 days or since the onset of the pain if pain duration was less than 7 days.^27^^,^^29^ Pain scores range from 0 to 10 (0 = no pain; 10 = worst pain imaginable).

Fear avoidance beliefs were assessed using the Fear-Avoidance Beliefs Questionnaire (FABQ).^30^ The FABQ score ranges from 0 to 96, and a higher score indicates stronger fear and avoidance beliefs regarding how physical activity affects LBP.

Pain catastrophizing was measured with the Pain Catastrophizing Scale (PCS).^31^ The PCS score ranges from 0 to 52, and a higher score on the PCS corresponds to a higher level of pain catastrophizing.

Self-efficacy was measured using the General Self-Efficacy Scale.^32^^,^^33^ The score ranges from 10 to 40, and a higher score corresponds to higher self-efficacy.

Self-management ability was rated using the Dutch language version of the short form Patient Activation Measure (PAM 13-Dutch).^34^ A higher score (range = 0–100) corresponds to a higher level of self-management.

Health-related quality of life was measured using the EuroQol-5D-5L.^35^ A higher score (range 0–1) corresponds with higher health-related quality of life.

### Data Analysis

Data preparation and calculation of descriptive statistics were performed using SPSS 27 (IBM Corp. Released 2020. IBM SPSS Statistics for Windows, Version 27.0, Armonk, NY) and R (R foundation, Vienna, Austria). Subsequent analyses were performed using R. For a longitudinal analysis of the data, at least 2 EXAS scores are required. The first EXAS score can be calculated after treatment session 2, based on patient adherence to HBE recommendations from the physical therapist given during the first session. Similarly, the second EXAS score can be calculated after the third treatment and so on. Therefore, data from patients with fewer than 2 EXAS scores were excluded. Missing values analyses were performed to evaluate if observed variables were correlated with variables with missing data. Relationships between baseline variables and missingness of adherence variables were found; therefore, further analyses of the data were performed by assuming data were missing at random. Multivariate imputation by chained equations was used to impute missing data in R using the mice package.^36^^,^^37^ One imputed dataset was created for every percent of cases with missing data for a total of 52 imputed datasets. To model latent class growth analysis (LCGA) trajectories using the imputed datasets, adherence LCGA trajectories were estimated in each separate imputed dataset. Second, all imputed datasets were used to create an “overall mean adherence trajectory.” This trajectory was obtained by pooling the mean adherence values at each follow-up moment over all patients and all imputed datasets. Then, the imputed dataset with the smallest mean difference from the overall mean adherence trajectory was selected and used for further analyses.

To assess the presence of subgroups of patients with distinct trajectories of adherence, LCGA was performed using the lcmm package in R.^38^ Trajectories were estimated for linear models and models with a quadratic term for time. Model fit was tested for solutions with 1, 2, 3, and 4 classes. To find the optimal model the maximum log-likelihood ratio, Akaike Information Criterion, Bayesian Information Criterion, and entropy values of the different models were compared.^39^ When less than 5% of the sample was assigned to a class, the model with k-1 classes was chosen instead to maintain the clinical usefulness of the final model. To test for differences in baseline characteristics between participants based on class membership from the LCGA, chi-square tests were used for categorical variables. The Kruskal–Wallis test was used for continuous variables due to the non-normal distribution of the data.

### Role of the Funding Source

The funder played no role in the design, conduct, or reporting of this study.

## Results

Data on adherence and received treatment during the study were available for 191 of the 208 participants. The unavailability of adherence data for a patient was caused by physical therapists not using the case report form properly during treatment or not returning the case report form to the researchers after the treatment ended. Eighteen patients received fewer than 3 treatments, leaving data from 173 participants available for analysis.

Data on patient adherence were collected during 5.1 (SD 2.5) treatment sessions and total treatment duration lasted for 51 (SD 41.7) days. Baseline characteristics of included patients can be found in Table 1. Results from the LCGA are limited to 11 treatment sessions (10 timepoints), because only 1 patient received more than 11 treatment sessions.

**Table 1:** Baseline Demographic and Clinical Characteristics for all Patients*^a^*

**Patient Characteristics**		***n* = 173**
Sex (female), *n* (%)		88 (50.9)
Age (y), median (IQR)		48 (24.3)
Height (cm), median (IQR)		175 (12)
Weight (kg), median (IQR)		80 (20)
BMI (kg/m^2^), median (IQR)		25.5 (4.7)
Educational level, *n* (%)		
	Low	30 (17.3)
	Middle	60 (34.7)
	High	83 (48.0)
Central sensitization (score = 0–100), median (IQR)		27 (18)
Duration of current LBP episode, *n* (%)		
	0–6 wk	72 (41.6)
	6–12 wk	26 (15.0)
	12 wk to 12 mo	15 (8.7)
	>12 mo	60 (34.7)
Physical functioning (score = 0–100), median (IQR)		18 (20)
Pain intensity (average score = 7 d, 0–10), median (IQR)		6 (3.0)
Fear-avoidance beliefs (score = 0–96), median (IQR)		23 (18)
Pain catastrophizing (score = 0–52), median (IQR)		8 (11)
Self-efficacy (score = 10–40), median (IQR)		33 (5)
Self-management ability (score = 0–100), median (IQR)		63.1 (19.3)
Health-related quality of life (score = 0–1), median (IQR)		0.9 (0.2)
Intervention group, *n* (%)		87 (50.3)

a BMI = body mass index; IQR = interquartile range; LBP = low back pain.

**Figure 1:**
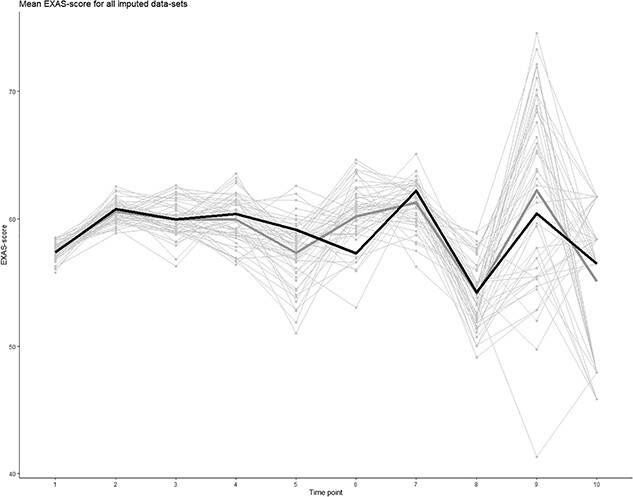
Overall mean adherence trajectory estimated from 52 imputed datasets (thick gray), mean adherence trajectory estimated from each of the 52 imputed datasets separately (thin gray), and mean adherence trajectory of the imputed dataset (#24) with the smallest mean deviation from the overall mean adherence trajectory (black).

Figure 1 shows the plotted overall mean values for the EXAS-score at each timepoint (thick gray line), plots for all individual imputed datasets (thin gray lines), and the plot for dataset #24 with the smallest mean deviation from the mean of all datasets (black line).

**Figure 2:**
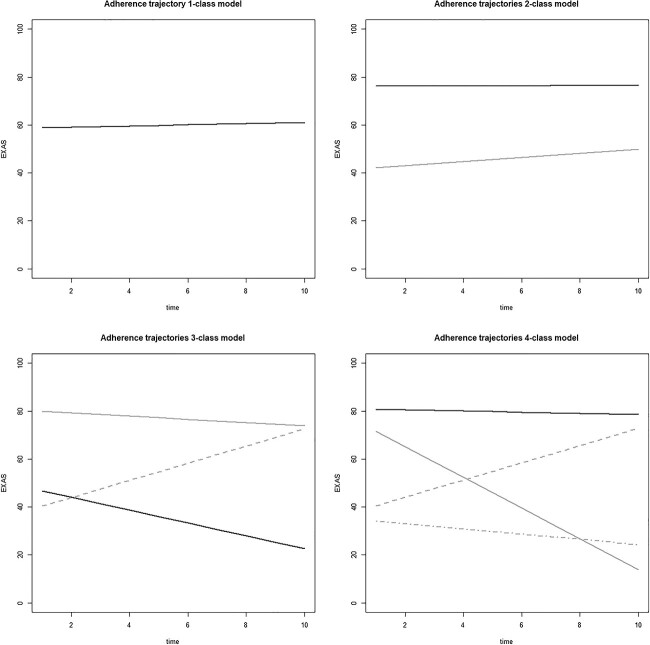
Predicted trajectories of adherence over time based on class assignment for the 1-class (black), 2-class (gray), 3-class (gray/dash), and 4-class (gray/dot-dash) trajectory models.

**Figure 3:**
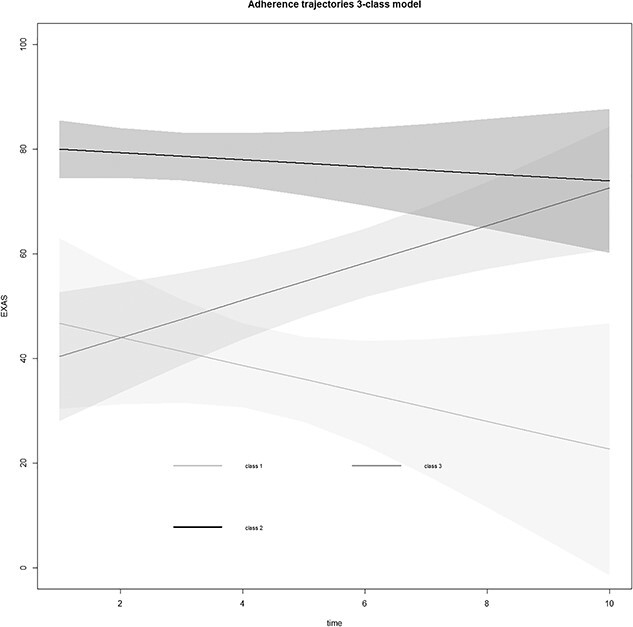
Estimated trajectories for the 3-class trajectory model with 95% CI.

Models with 1 to 4 classes were estimated using LCGA. The addition of a quadratic term for time did not increase the fit of the linear models. The 4-class solution showed optimal performance based on the maximum log-likelihood criterion, with a value of −3115.1, while the 2-class and 3-class models yielded lower log-likelihood scores of −3126.3 and − 3119.1, respectively. Similarly, the Akaike Information Criterion favored the 4-class solution with a value of 6254.2, compared to the 2-class (6264.7) and 3-class (6256.3) models. Conversely, the Bayesian Information Criterion favored the 2-class model with a lower value of 6283.6, in contrast to the 3-class (6284.6) and 4-class (6292.0) models. Furthermore, the entropy measure indicated a better fit for the 2-class model (0.61) compared to the 3-class (0.49) and 4-class (0.54) models. However, the 2-class model displayed 2 nearly parallel trajectories (Fig. 2), suggesting limited clinical significance and within the 4-class model, the fourth class contained less than 5% (4.6%) of the patient population. Therefore, the k-1 model (model 3) was chosen instead (Fig. 3).

Baseline characteristics of the patient groups for each trajectory class are shown in Table 2. Approximately 12% of participants belong to the “declining adherence” class, 45% to the “stable adherence” class, and 43% to the “increasing adherence” class. No differences were found between the 3 patient groups based on baseline characteristics. Additionally, no differences in the proportion of patients from the treatment group in the parent trial were observed between the trajectory classes.

**Table 2:** Comparison Between Identified Trajectory Classes Based on Baseline Characteristics*^a^*

**Patient Characteristics**		**Class 1 Low Declining Adherence (*n* = 21)**	**Class 2 High Stable Adherence (*n* = 78)**	**Class 3 Low Increasing Adherence (*n* = 74)**	***P* Between Groups**
Age (y), median (IQR)		45 (17.8)	47.7 (25.5)	49.1 (21.2)	.78
Height (cm), median (IQR)		173 (10)	175 (12.8)	174.5 (14)	.83
Weight (kg), median (IQR)		80 (12)	77.5 (22.8)	79 (22)	.68
BMI (kg/m^2^), median (IQR)		25.1 (5.4)	24.9 (4.7)	26 (4.9)	.32
Central sensitization (score = 0–100), median (IQR)		31 (25)	26 (15.8)	29 (18)	.29
Physical functioning (score = 0–100), median (IQR)		22 (26)	18 (16)	18 (20)	.84
Pain intensity (average score = 7 d, 0–10), median (IQR)		6 (3)	6 (3)	6 (3)	.49
Fear-avoidance beliefs (score = 0–96), median (IQR)		25 (23)	20.5 (18)	24.5 (17)	.20
Pain catastrophizing (score = 0–52), median (IQR)		12 (9.0)	8 (10)	8.5 (13.5)	.51
Self-efficacy (score = 10–40), median (IQR)		32 (5)	34 (5.8)	32 (5.8)	.79
Self-management ability (score = 0–100), median (IQR)		63.1 (14.6)	63.1 (16.9)	63.1 (19.3)	.78
Health-related quality of life (score = 0–1), median (IQR)		0.85 (0.2)	0.89 (0.2)	0.89 (0.2)	.46
Sex (female), *n* (%)		10 (47.6)	43 (55.1)	35 (47.3)	.60*^b^*
Educational level, *n* (%)					.78*^b^*
	Low	5 (23.8)	15 (19.2)	10 (13.5)	
	Middle	7 (33.3)	25 (32.1)	28 (37.8)	
	High	9 (42.9)	38 (48.7)	36 (48.6)	
Duration of current LBP episode, *n* (%)					.62^b^
	0–6 wk	9 (42.9)	29 (37.2)	34 (45.9)	
	6–12 wk	4 (19.0)	13 (16.7)	9 (12.2)	
	12 wk to 12 mo	1 (4.8)	5 (6.4)	9 (12.2)	
	>12 mo	7 (33.3)	31 (39.7)	22 (29.7)	
Intervention group, *n* (%)		12 (57.1)	41 (52.6)	34 (45.9)	.57*

^a^ % = percentage of the total sample; BMI = body mass index; IQR = interquartile range; LBP = low back pain.

^b^ *P* from chi-square test.

## Discussion

The current study aimed to investigate the presence of groups of patients with distinct trajectories of adherence to HBE recommendations among people with LBP and to identify differences in baseline characteristics between groups. Three groups with distinct trajectories were identified. The “low declining adherence” group started with moderate adherence and declined to almost no adherence over the course of treatment. The “low increasing adherence” group started at around the same level of moderate adherence as the “low declining adherence” group, but adherence increased over time to almost 80 points on the EXAS. The “high stable adherence” group started with the highest adherence, and adherence declined slowly to approximately the same level as the “low increasing adherence” group at the end of the trajectory. None of the baseline characteristics showed statistically significant differences between the identified trajectory classes, including treatment group allocation in the intervention study. It is noteworthy that the width of the confidence intervals of the trajectories increases sharply as the number of treatments increases. This is because the number of patients still receiving treatment declines quickly after 6 treatment sessions reducing the precision of estimated trajectories past this point. To our knowledge, the current study is the first to measure adherence trajectories to HBE recommendations in patients with LBP during treatment by a physical therapist, making direct comparison of our results with similar studies in patients with LBP difficult. However, trajectories of adherence were previously investigated in patients with osteoarthritis of the knee and/or hip.^17^ The authors found 3 distinct trajectories of adherence over time, similar to the current study. A major difference with the current study, however, is the development of the identified trajectories over time. In patients with osteoarthritis of the knee and/or hip, the trajectories either declined gradually or rapidly, or adherence was low for the entire trajectory. This contrasts with the trajectories found in the current study, which started either around the 40-point mark or at the 80-point mark with a gradual change over time and only the trajectory for the smallest group (12.1% of the participants) showed a large decrease in adherence over time. The patients belonging to the other groups reported either increasing adherence or very slowly decreasing adherence over the course of treatment, with both groups ending up at roughly the same level of adherence after 10 treatment sessions. A possible explanation for this difference between the trajectories of adherence found in both studies is the time period over which the measurements were taken. In our study, all treatments ended within 12 weeks and measurements were only taken while the patient was still being treated by their therapist, whereas in the other study results were included from studies where treatment lasted from 12 weeks to 6 months and adherence was measured for 36 to 78 weeks. As a result, patients in our study most likely had far more opportunities to receive support from their therapist during the period in which adherence was measured, leading to higher adherence numbers. Furthermore, the longer time period during which measurements were taken in the study with patients with osteoarthritis of the hip and/or knee allows for more time for adherence to decline, resulting in a higher likelihood of decreasing adherence over time. Another explanation is the difference in measurement instruments used to measure adherence between the studies. The EXAS used in the current study provides a more accurate measurement of adherence than the recall over several weeks used in the other study.

In patients with osteoarthritis of the knee and/or hip, differences between the identified groups were found for pain, function, and self-efficacy. This is in line with studies investigating factors associated with adherence but is in stark contrast to the findings from the current study.^8^ Despite the fact that baseline characteristics were selected for the comparison based on existing literature,^8^^,^^10^ none of the baseline characteristics measured were significantly different between the trajectory groups in our study. There are several possible explanations for the differences between factors related to adherence found in the literature and the findings of the current study. The first and most straightforward explanation is that patient adherence to HBE recommendations during treatment is determined by patient characteristics that were not measured and therefore no differences between groups could be found. However, the baseline characteristics chosen for baseline comparison between groups were carefully selected based on existing literature and have consistently been shown to be related to adherence. This makes it unlikely that a single patient characteristic explaining the different trajectories was missed and left out of the analysis.

Another explanation is that adherence to HBE recommendations during treatment is mainly determined by factors outside of the patient, such as environmental factors, social factors, intervention-related factors, or therapist-related factors. Indeed, a number of the factors related to adherence reported in the literature are external factors not directly related to the patient.^8^^,^^10^ For instance, a recent pilot study showed very high adherence when patients received external support in the form of telemonitoring and regular check-ups from their physical therapist.^40^ However, this would mean that external factors are far more important than patient factors for patient adherence during treatment. Although possible, it seems unlikely that patients have little influence on their own adherence to HBE recommendations during physical therapist treatment. A more plausible explanation is that adherence to HBE recommendations is not determined by baseline patient characteristics alone, but also by the change in these characteristics over time as treatment progresses and interactions between patients, their environments, and their physical therapists. For example, a physical therapist can incorporate strategies to support or increase self-efficacy in patients with low self-efficacy at the start of treatment in an attempt to increase adherence during treatment. For future research, it would be interesting to combine repeated measurements of baseline characteristics with measurement of adherence. Combined with investigating the patient-therapist interactions during treatment sessions and their effects on patient adherence, this can help to further understand patient adherence.

### Strengths and Limitations

Our study has a number of strengths. The application of multiple imputation by chained equations for missing data helped to reduce bias introduced by missing data. Since a large number of cases had at least 1 missing data point due to illegible reporting by the therapist, no reporting by the therapist or other problems not related to the patient, performing a complete case analysis would have significantly reduced the number of cases available for the analysis. Imputing missing data allowed optimal use of the available data and therefore provide more robust results. Another strength of the study is the use of the EXAS for the measurement of adherence during every treatment session. The detailed information on patient adherence provided by the EXAS allowed the use of LCGA to determine different groups of patients with distinct adherence trajectories.

Limitations of the study should also be discussed. The first limitation is the introduction of missing data through the way data on adherence was collected. To keep the added workload for the physical therapists participating in the study low, we chose a method that allowed the physical therapists to write down the data on a form they could keep on their desk. Although this methodology requires little effort from the therapist, it introduced more room for errors in reporting (illegible handwriting, forgetting to complete part of the form, etc.) than for example digital reporting through a web–based application. Although imputation was used to minimize the effects of missing data on the results, the best way to handle missing data is to prevent it. A second limitation is that there are currently no existing rules or conventions for the pooling of estimates from LCGA on imputed datasets. Imputation of missing data and analysis of the imputed data generally consists of 3 steps.^37^ First, a number of different datasets with imputed data are created. Then, the parameters of interest are estimated from each imputed dataset. The last step is the pooling of the parameter estimates and estimating the variance of the pooled estimate. Although the mice package from R provides the tools to pool estimates for linear models, these tools are not available for LCGA in the mice package. Although manually pooling and estimating the parameters of interest would have been possible, similar procedures for the Kruskal-Wallis test used to compare baseline characteristics of the identified trajectory groups do not exist. Instead, we decided to calculate the average of all variables with imputed data over all imputed datasets and find the dataset with the smallest mean deviation from the overall mean to perform the analyses on. This allows the use of imputation to maximize the data available for analysis at the cost of precision of the estimated parameters and estimated variance. The last limitation of the study is the higher proportion of patients with a duration of the current episode of LBP of less than 12 weeks is greater than the proportion of patients with a longer duration of LBP at the start of the study. This difference in proportions might make it difficult to generalize the current findings to patients with chronic LBP. However, the proportions of patients with a duration of the current episode of more than 12 weeks are roughly similar between all 3 trajectory classes at 38.1, 46.1, and 41.9%, respectively. Furthermore, these proportions are again roughly similar to the proportion of 43.4% of patients with a duration of the current episode of more than 12 weeks in the entire sample. Therefore, it appears that the results from the current study can be reasonably well generalized to patients with LBP of all durations.

Although no differences between baseline characteristics of the identified trajectory groups were found, the results show that there is no single trajectory of adherence for all patients and that it might not be possible to distinguish different subgroups based on baseline characteristics alone. Therefore, when planning patient treatment, clinicians should not attempt to determine adherence of their patients at the start of treatment and base interventions on that assessment. Instead, monitoring adherence during treatment using an instrument such as the EXAS and intervening when adherence is too low appears to be the optimal strategy.

Future research should incorporate the patient–therapist interaction, the patient’s social environment, and patient characteristics when studying patient adherence to better understand how patient adherence can be supported during physical therapist treatment. Another important next step in the research on patient adherence in patients with LBP is to study the association between trajectories of adherence to HBE and clinical outcomes to assess the effects of adherence on clinical outcomes.

## Conclusion

Three different trajectories of adherence to HBE recommendations were identified in patients with LBP. No differences in baseline characteristics were found between the 3 trajectory groups; therefore, physical therapists should not attempt to place a patient in a trajectory group at the start of treatment. Instead, adherence should be closely monitored as treatment progresses and supported when required.

## Data Availability

The datasets generated, analyzed, or both during the current study will be available from the corresponding author on reasonable request on the completion of the study.
